# Fabrication of a Polyaniline Ultramicroelectrode via a Self Assembled Monolayer Modified Gold Electrode

**DOI:** 10.3390/s130708079

**Published:** 2013-06-24

**Authors:** Gulcin Bolat, Filiz Kuralay, Gunes Eroglu, Serdar Abaci

**Affiliations:** 1 Department of Chemistry, Faculty of Science, Hacettepe University, Beytepe-Ankara 06800, Turkey; E-Mails: gbolat@hacettepe.edu.tr (G.B.); geroglu@hacettepe.edu.tr (G.E.); 2 Department of Chemistry, Ordu University, Ordu 52200, Turkey; E-Mail: filizkur@hacettepe.edu.tr or kuralay.filiz@gmail.com

**Keywords:** polyaniline, self-assembled monolayer (SAM), microelectrode, gold electrode, DNA

## Abstract

Herein, we report a simple and inexpensive way for the fabrication of an ultramicroelectrode and present its characterization by electrochemical techniques. The fabrication of polyaniline UME involves only two steps: modification of a gold (Au) electrode by self assembled monolayers (SAM) and then electrodeposition of polyaniline film on this thiol-coated Au electrode by using cyclic voltammetry and constant potential electrolysis methods. Two types of self-assembled monolayers (4-mercapto-1-butanol, MB, and 11-mercaptoundecanoic acid, MUA) were used, respectively, to see the effect of chain length on microelectrode formation. Microelectrode fabrication and utility of the surface was investigated by cyclic voltammetric measurements in a redox probe. The thus prepared polyaniline microelectrode was then used for DNA immobilization. Discrimination between double-stranded DNA (dsDNA) and single-stranded DNA (ssDNA) was obtained with enhanced electrochemical signals compared to a polyaniline-coated Au electrode. Different modifications on the electrode surfaces were examined using scanning electron microscopy (SEM).

## 1. Introduction

Ultramicroelectrodes (UMEs), having tip dimensions of the order of a micrometre (1 μm) and even one tenth of a μm, have been used in various voltammetric studies in the last decade. The electrochemical behaviour of these ultramicroelectrodes is different from that of classical microelectrodes and they have major advantages in analytical applications [1–5]. UMEs have higher signal-to-noise ratios because the analytically relevant Faraday currents are greatly enhanced and this enhancement results from the hemispherical or spherical (rather than planar) diffusion towards UMEs [6,7].

UMEs have been fabricated by many different technologies (plasma enhanced chemical vapor deposition (PECVD), screen printing processes, photolithography, assembly of microdisks connected in parallel series, *etc.*) using a variety of materials (platinum, iridium, carbon fiber, and gold) with different shapes [8]. However, these processes require expensive instrumentation, using substrates such as wafers or Si/SiO_2_/metal substrates and also a trained staff.

Compared with other techniques, electrochemical manufacturing is favored because of its low cost and simplicity. In this contex, we propose for the first time a simple method for the design of polyaniline ultramicroelectrodes using electrochemical techniques. This method involves two steps: the first stage is to form a self-assembled monolayer (SAM) on the surface. The properties of self-assembled monolayers which are composed of organic molecules and terminated with various functional groups, have attracted a lot of interest in recent years [9]. These well-ordered and densely packed self-assembled monolayers (SAMs) have been used to modify the chemical and electrochemical properties of substrates for the deposition of films. SAMs of functionalized adsorbates on solid substrates generate well-defined organic surfaces with useful and highly alterable chemical functionalities which can provide a means to control the interface at a molecular level and exhibit very specific molecular properties [10–14]. The gate sites within a film which is free of pinhole defects, can act as a platform for 2D surface-confined polymerization of monomers parallel to the surface [15,16]. SAM-modified surfaces are suitable to use as electronic and organic semiconductors, electrochromic materials and for photocatalytic applications [17,18]. Functionalized SAMs were also used to modify the chemical response of surfaces in contact with solutions, to immobilize redox-active probes onto electrode surfaces in order to study electron-transfer reactions [19,20].

In this work the second stage for the UME construction was to deposit a conducting polymer, polyaniline (PANI), on the SAM-modified surface. Electrochemical deposition of conducting polymers on electrodes covered with thiol films can lead to the preparation of ultrathin polymer-thiol layers [10,21,22]. PANI has been studied quite extensively because of its useful functions such as a well known redox activity, high conductivity and stability under ambient conditions [23–25]. It can be formed either by chemical or electrochemical methods. Rubinstein *et al.* showed that PANI-coated 4-ATP-modified gold electrodes had better electrochemical characteristics than bare electrodes [26]. The effect of the monolayer structure on the electrochemical deposition of polyaniline and polypyrrole on gold substrates modified with various *w*-substituted alkanethiols was studied by Collard *et al.* [27]. Also, electrochemical deposition of conducting polymer films of poly(2-methoxyaniline) and poly-2,5-dimethoxyaniline on thiol-coated electrodes containing pinhole defects was investigated and the microelectrode behaviour was studied by Mazur *et al.* by application of the appropriate potential on the electrode [28].

The determination of DNA is vital for various applications such as genetic mutations, drug discovery, environmental science, forensics and food technology [29,30]. DNA can be immobilized onto different platforms for the development of novel sensors by adsorption, entrapment, complexation, covalent attachment, and other methods [31,32]. Electrochemical transducers such as polymer-modified electrodes can alternatively provide a suitable interface for the immobilization of DNA [33–35].

In this context, we propose a simple method for the design of polyaniline ultramicroelectrodes by using electrochemical techniques for the first time. Aniline was electropolymerized on thiol-coated gold electrodes to get polyaniline ultramicroelectrodes. The electrochemical behaviour of the as-formed electrodes was investigated using the cyclic voltammetry (CV) technique. UME formation was verified by cyclic voltammograms in redox probe and scan rate studies. DNA immobilization on the modified electrodes were performed and enhanced electrochemical signals were obtained compared to the results obtained with only polyaniline-coated electrodes. Discrimination of double-stranded DNA (dsDNA) and single-stranded DNA (ssDNA) was carried out sensitively using UME. Different modifications on the electrode surfaces were identified using scanning electron microscopy (SEM).

## 2. Experimental

### 2.1. Chemicals and Reagents

Aniline (99.9%), HClO_4_, H_3_PO_4_, NaH_2_PO_4_.2H_2_O, Na_2_HPO_4_·2H_2_O and NaOH were obtained from Merck (Darmstadt, Germany). 11-Mercaptoundecanoic acid (MUA) was purchased from Sigma-Aldrich (Steinheim, Germany) and 4-mercapto-1-butanol (MB) was purchased from Fluka (Steinheim, Germany). KCl was obtained from Fisher (Guangdong, China). K_3_Fe(CN)_6_ and K_4_Fe(CN)_6_ were obtained from J.T. Baker (Phillipsburg, NJ, USA). Calf thymus double-stranded and single-stranded DNA (ds/ssDNA) was purchased from Sigma. Other chemicals were in analytical reagent grade and were obtained from Sigma or Merck.

### 2.2. Instrumentation

All electrochemical experiments were carried out using a CH Instruments (USA) model CHI660C potentiostat connected to a personal computer to perform cyclic voltammetric and bulk electrolysis studies. The morphologies of the obtained films were examined using a Zeiss Evo 50 EP-SEM Model Scanning Electron Microscope (SEM, USA). A standard three electrode cell comprising a Au electrode with an area of 0.0314 cm^2^ as a working electrode, a Ag/AgCl reference electrode and a Pt wire counter electrode was used. The working electrode was polished with 0.05 μm and 0.1 μm alumina paste before each analysis.

### 2.3. Preparation of Solutions

**Figure 1.**
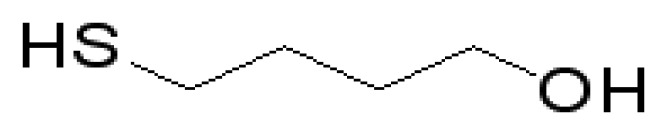
4-Mercapto-1-butanol (MB).

**Figure 2.**
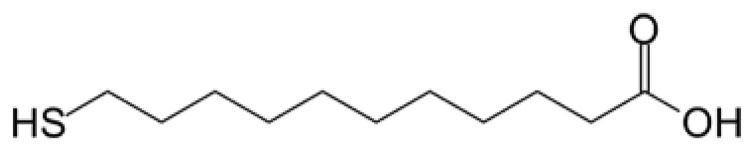
11-Mercaptoundecanoic acid (MUA).

4-Mercapto-1-butanol (Figure 1) and 11-mercaptoundecanoic acid (Figure 2) solutions were prepared with ethanol. Phosphate buffer solution (50 mM PBS, pH: 7.0) was prepared from NaH_2_PO_4_·2H_2_O and Na_2_HPO_4_·2H_2_O using triple distilled water. 0.1 M Fe(CN)_6_^3-/4-^ solutions containing 0.1 M KCl were prepared using K_4_Fe(CN)_6_ and K_3_Fe(CN)_6_. ds/ssDNA stock solutions were prepared with ultrapure tri-distilled water and kept frozen. The diluted solutions of adenine were prepared by using PBS containing 20 mM NaCl. All the solutions were purged with N_2_ gas (BOS, 99.99%) for 10 min prior to measurements.

### 2.4. Procedures

Thiol monolayers were formed on Au substrates by immersing the electrodes in ethanol solution containing 0.1 M of MB (Figure 1) or MUA (Figure 2) for 20 minutes and rinsing with ethanol solution. Then, the thiol modified electrodes were coated with polyaniline (PANI) by performing cyclic voltammetry (scanning between +0.0 V and +1.4 V *vs.* Ag/AgCl, scan rate: 50 mV/s) and bulk electrolysis (at +0.9 V *vs.* Ag/AgCl) in 0.1 M monomer solutions in the presence of 0.1 M HClO_4_ according to a previously reported procedure [36]. DNA immobilization on the electrodes was performed by immersing the working electrodes in ds/ssDNA solutions for 30 min. The electrodes were washed using buffer solution after DNA immobilization. The differential pulse voltammetry (DPV) technique was applied between +0.4 and 1.4 V *vs.* Ag/AgCl at pulse amplitude of 50 mV in 50 mM PBS.

## 3. Results and Discussion

**Figure 3.**
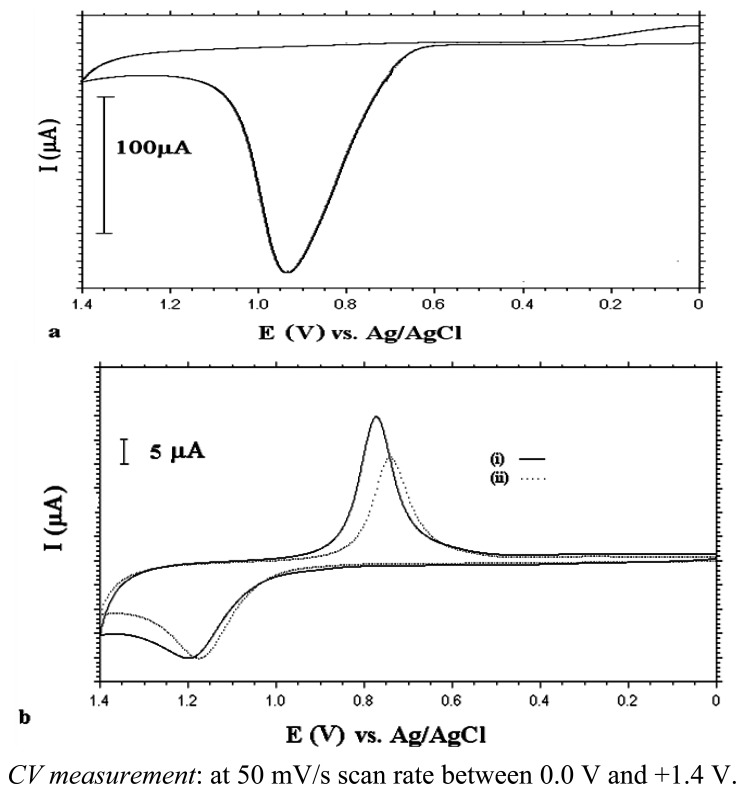
(**a**) CV of 0.1 M aniline in 0.1 M HClO_4_ at MB modified Au electrode (first scan) (**b**) CVs of (i) bare Au electrode (ii) MB modified Au electrode in 0.1 M HClO_4_.

The planned strategy on UME production was to modify a bare Au electrode with a self-assembled monolayer and then coat it with a conducting polymer. Two types of SAMs were used. First, 4-mercapto-\1-butanol (MB) was coated as described in the Experimental section. The second step was to coat this modified surface with polyaniline. Figure 3(a) shows the electrochemical behaviour of aniline on this modified surface. As can be seen from this figure, aniline was oxidized at around +0.94 V *vs.* Ag/AgCl using the MB-coated Au electrode. The thiol group didn't oxidize between 0.0 V and +1.4 V *vs.* Ag/AgCl potential interval. It was stable and didn't reveal desorption from the surface (Figure 3(b-ii)).

Different polymerization methods can be applied for polyaniline formation. However, the critical point for aniline polymerization is to form polymer islands on surface defects of the MB-modified Au surface which can act as microelectrodes.

Electropolymerization of aniline was accomplished by two different methods. In the cyclic voltammetric (CV) method, the potential was scanned between 0.0 V and +1.4 V for 10 cycles. The sharp oxidation peak observed in the first cycle of aniline electropolymerization (Figure 3(a)) indicates electropolymerization of aniline proceeds by nucleation through the defect sites of SAMs.

**Figure 4.**
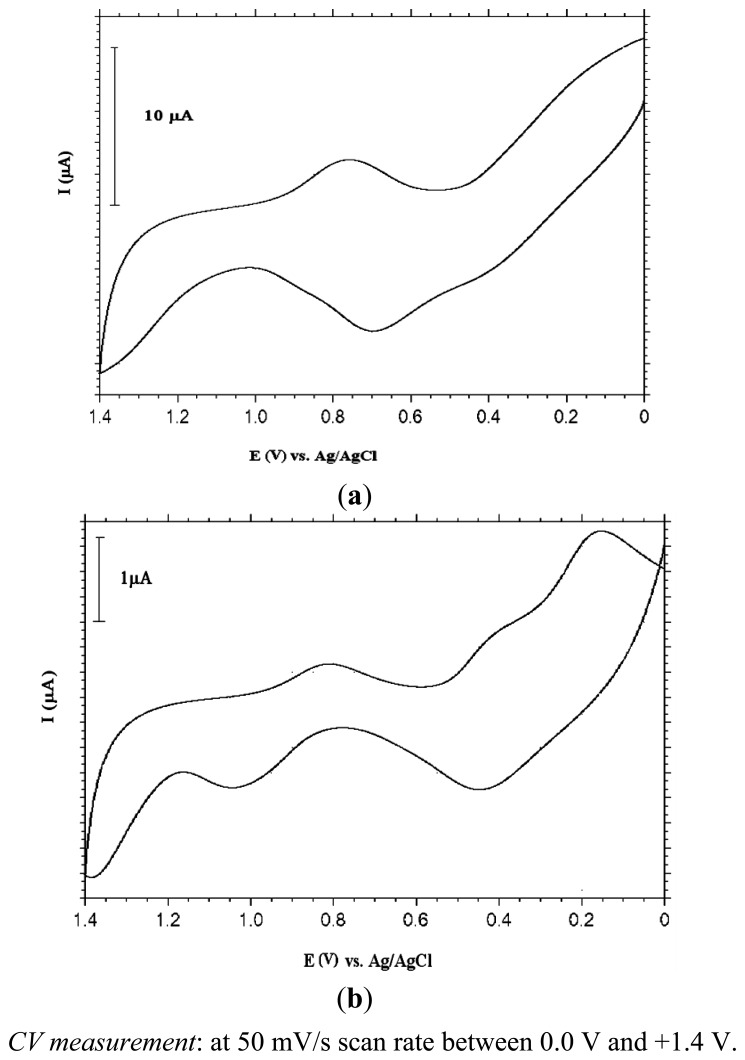
(**a**) CV of PANI (deposited by CV method: 10 cycles) coated MB modified Au electrode in blank solution (0.1 M HClO_4_) (**b**) CV of PANI (deposited by bulk electrolysis: at 0.9 V for 20 minutes) coated MB modified Au electrode in blank solution (0.1 M HClO_4_).

After the polymerization step, the electrode was inserted into a blank solution (0.1 M HClO_4_) to check polymer formation. Depending on the degree of oxidation, electrochemically prepared PANI exists in different forms, such as leucoemeraldine, emeraldine and pernigraline. Leucoemeraldine base refers to the fully reduced form, emeraldine base is half-oxidized, while pernigraline is a completely oxidized form of polyaniline [37]. As can be seen from Figure 4(a), PANI film deposited by using CV, was in mostly oxidized form of aniline (pernigralin) and no degradation due to overoxidation appeared [36,38].

Constant potential electrolysis was also used for PANI formation. Electrolysis was carried out at +0.9 V *vs.* Ag/AgCl for 20 minutes. After electrolysis, the electrode was inserted into 0.1 M HClO_4_ to check polymer formation. As seen from Figure 4(b), PANI was in partially oxidized and reduced forms [38]. The first well-defined peak at +0.4 V indicates oxidation of the leucoemeraldine form of polyaniline to the emeraldine salt and the second anodic peak situated at +1.0 V corresponds to the oxidation of emeraldine to the pernigraline form. On the reverse scan two reduction peaks corresponding to these peaks were observed.

As mentioned above, the aim was to get UMEs on a SAM-covered Au surface. To test whether this was accomplished or not, the electrochemical behaviour of PANI-deposited MB-modified electrodes was investigated in Fe(CN)_6_^3−/4−^ solution in parallel to the reported studies [16,39] since Fe(CN)_6_^3−/4−^ has well-known redox behaviour. As known, classical microelectrodes give sinusoidal shaped voltammograms, whereas the ultramicroelectrode response is S-shaped [7,39–41]. Surface defects in the SAM leads to 2-D polymerization instead of 3-D growth on the electrode surface. As a result, a sigmoidal-shaped voltammogram should have been obtained due to mass transport through radial diffusion to defect sites.

**Figure 5.**
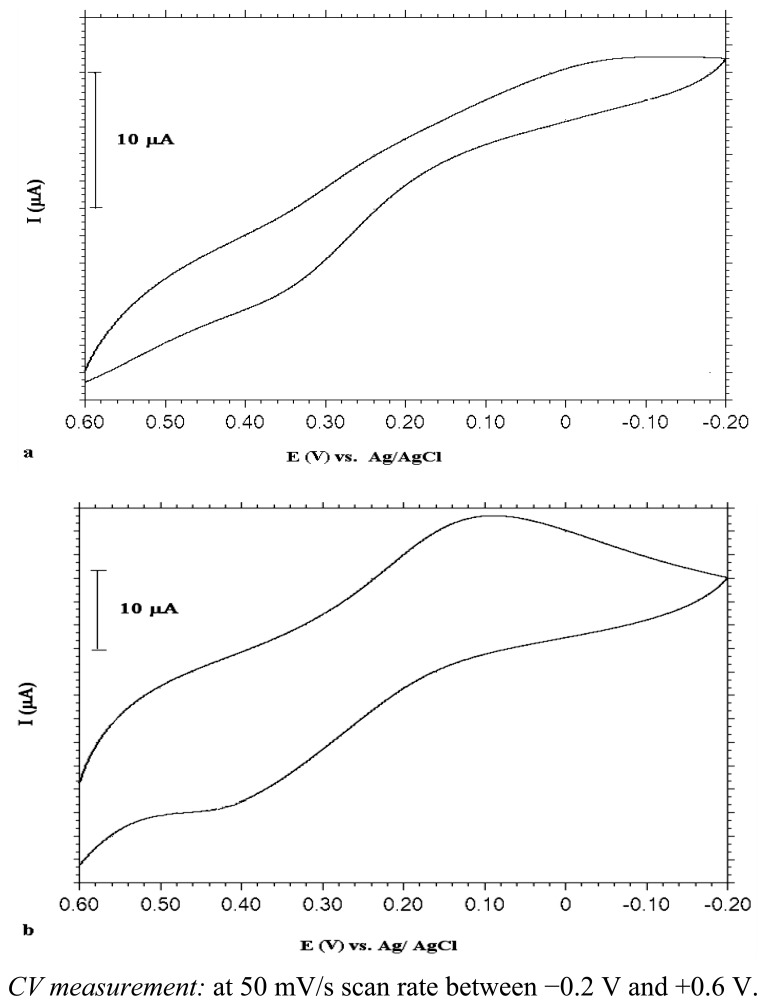
CVs of PANI (**a**) (deposited by CV method: 10 cycles) (**b**) (deposited by bulk electrolysis: at 0.9 V for 20 minutes) coated MB modified Au electrode in 0.1 M Fe(CN)_6_^3−/4−^ solution.

Figure 5 shows the responses in Fe(CN)_6_^3−/4−^ solution. As can be deduced from Figure 5(a), the surface which was formed by depositing PANI by CV method onto a 4-mercaptobutanol-modified gold surface (UME-1) gave almost S-shaped voltammograms, however, the constant potential method led to non-UME behaviour exhibiting peak currents due to diffussional mass transport limitations on the surface (Figure 5(b)). These results can be explained as follows: in the constant potential electrolysis method, polymeric films were distributed inhomogeneously on the MB-modified gold surface due to a fast polymer growth rate, but the CV method provided better polymeric film distribution with each polymerization cycle due to nucleation.

**Figure 6.**
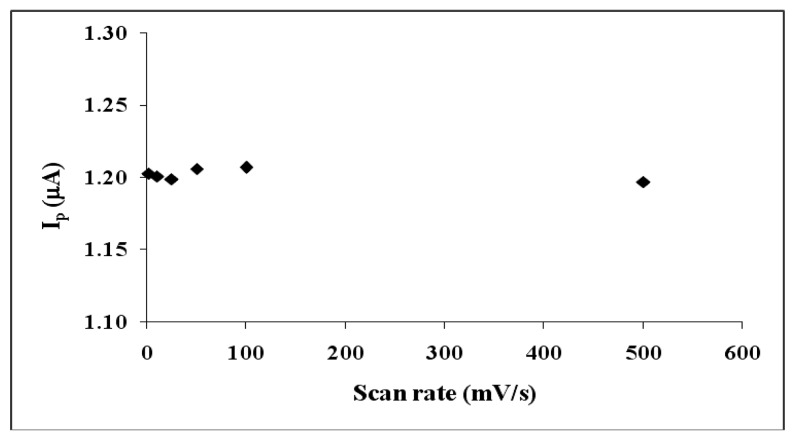
The effect of scan rate using UME-1 (at 2, 10, 25, 50, 100 mV/s).

Scan rate studies were also carried out to verify the ultramicroelectrode formation. The results of this study for UME-1 is shown in Figure 6. As expected, peak current did not change much with low scan rates. This was another indication of UME formation [39].

**Figure 7.**
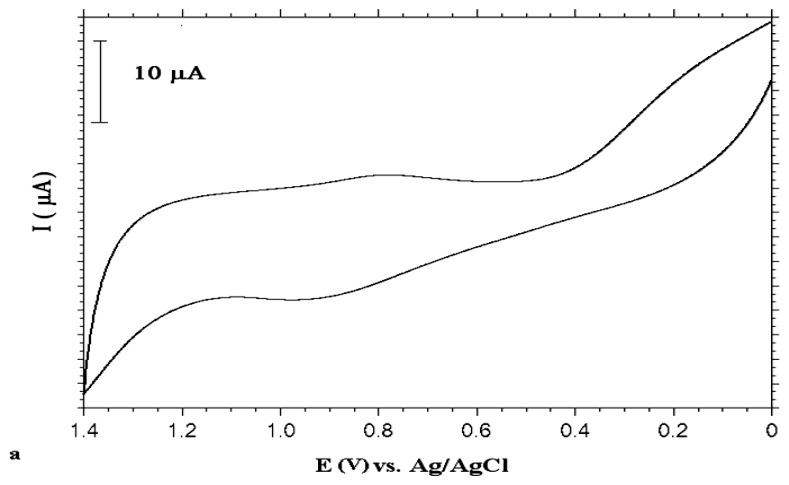

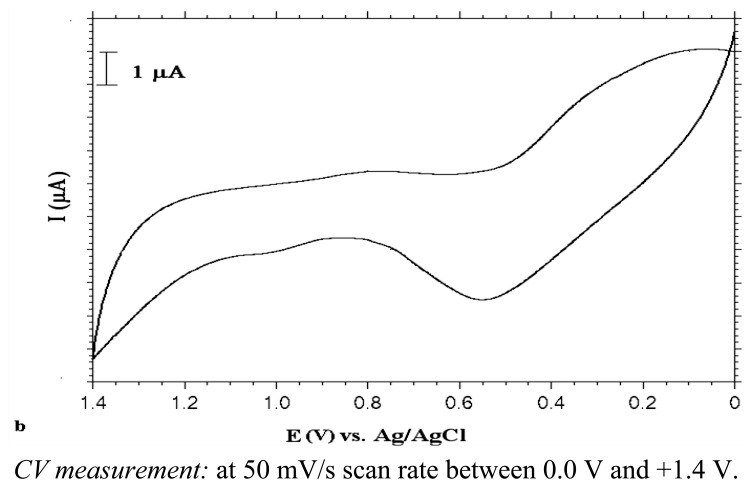
(**a**) CV of PANI (deposited by CV method: 10 cycles) coated MUA modified Au electrode in blank solution (0.1 M HClO_4_) (**b**) CV of PANI (deposited by bulk electrolysis: at 0.9 V for 20 minutes) coated MUA modified Au electrode in blank solution (0.1 M HClO_4_).

It has been reported that polymer formation is higher for a monolayer comprising hydrophilic (hydroxyl, carboxy) rather than hydrophobic (methyl) terminal groups by facilitating the penetration of hydrophilic monomers such as aniline [16]. In order to determine the effect of type and chain length of self assembled monolayer on UME fabrication, another thiol molecule, 11-mercaptoundecanoic acid (MUA) was used. This molecule was coated on a bare Au electrode as was described in the Experimental section. Polymerization of aniline was also carried out with the CV and constant potential electrolysis methods, respectively. Blank solution voltammograms for each case are shown in Figure 7. Lesser amounts of bulk polymer and thus an increased number of polymer islands were obtained compared to the MB-assembled gold surface. The defects formed by the long chained monolayer of MUA facilitated nucleation and growth of PANI microstructures. Blank solution (0.1 M HClO_4_) of PANI deposited by the CV method on a MUA-modified Au electrode showed that the polymer obtained was in its conducting, oxidized form and was expected to behave as a microelectrode. In order to see this, the response of the electrodes were examined in redox probe solution.

**Figure 8.**
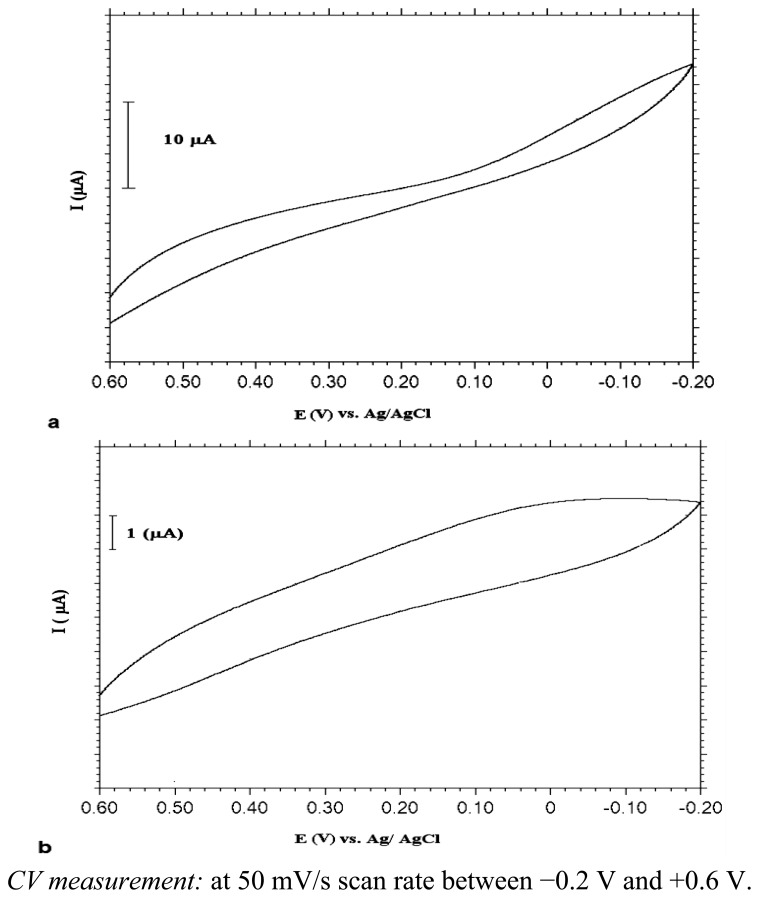
CVs of PANI (**a**) (deposited by CV method: 10 cycles) (**b**) (deposited by bulk electrolysis: at 0.9 V for 20 minutes) coated MUA modified Au electrode in 0.1 M Fe(CN)_6_^3−/4−^ solution.

Figure 8 shows the electrochemical responses of PANI deposited by the CV and bulk electrolysis methods, respectively, on MUA-modified Au electrodes in Fe(CN)_6_^3−/4−^ solution. Based on these voltammograms, it can be claimed that ultramicroelectrode fabrication was achieved with polyaniline that was formed with the CV method on the MUA-modified Au surface (UME-2). However, there was no noticeable electrochemical activity with the polyaniline film obtained with the constant potential electrolysis method.

When compared with the voltammogram obtained with PANI on MB-modified Au (Figure 5(a)), it is clear that UME behaviour of the electrode modified with thiol molecule, MUA, having a carboxylic acid end group and a longer hydrocarbon chain length is better. The shape of the voltammogram in the redox probe resembled UME behaviour much more. Non-linear diffusion caused by regional conduction of polymer islands is responsible for this sigmoidal shape of CV curve.

**Figure 9.**
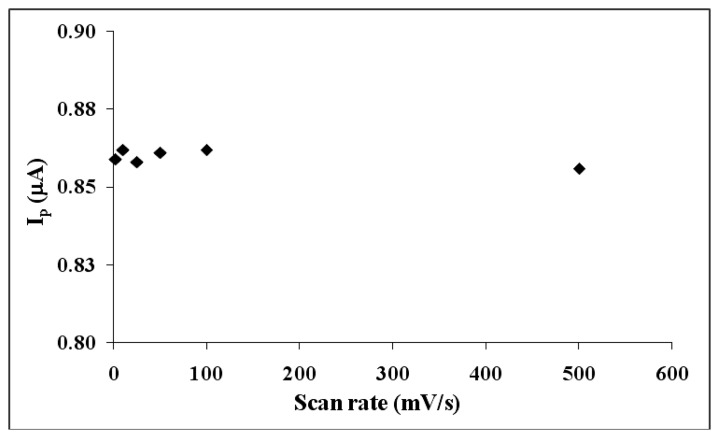
The effect of scan rate using UME-2 (at 2, 10, 25, 50, 100 mV/s).

In order to check the ultramicroelectrode formation, scan rate studies were also carried out and shown in Figure 9. According to this figure, peak current did not change with low scan rates. This was another indication of UME formation in parallel to the literature [1–8]. These results revealed that PANI film on MUA-modified gold prepared by CV gave the best UME-like behaviour response.

**Figure 10.**
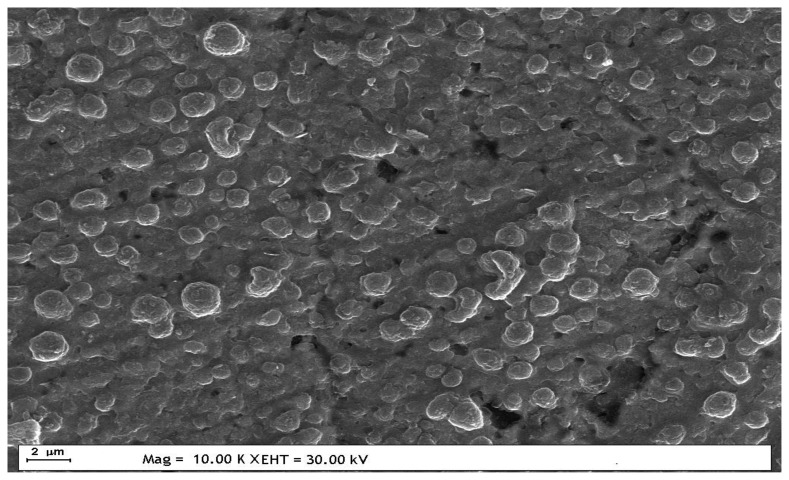
SEM image of PANI deposited (CV method) on a MUA-modified Au electrode.

SEM images of the film which was formed by polyaniline with the CV method on a MUA-modified gold electrode were recorded since this film showed the best UME behaviour with electrochemical methods. As can be seen from Figure 10, PANI nuclei were distributed almost homogenously onto the defect sites of the electrode surface. The observed characteristic globular structure of the polymer islands can be ascribed to the presence of 2D growth during polymerization. The average diameter of the polymer nuclei is ca. 0.8–1.2 μM. These results are in agreement with the CV investigations revealing radial diffusion to the electrode surface and causing sigmoidal shaped voltammograms in Fe(CN)_6_^3−/4−^ solution.

**Figure 11.**
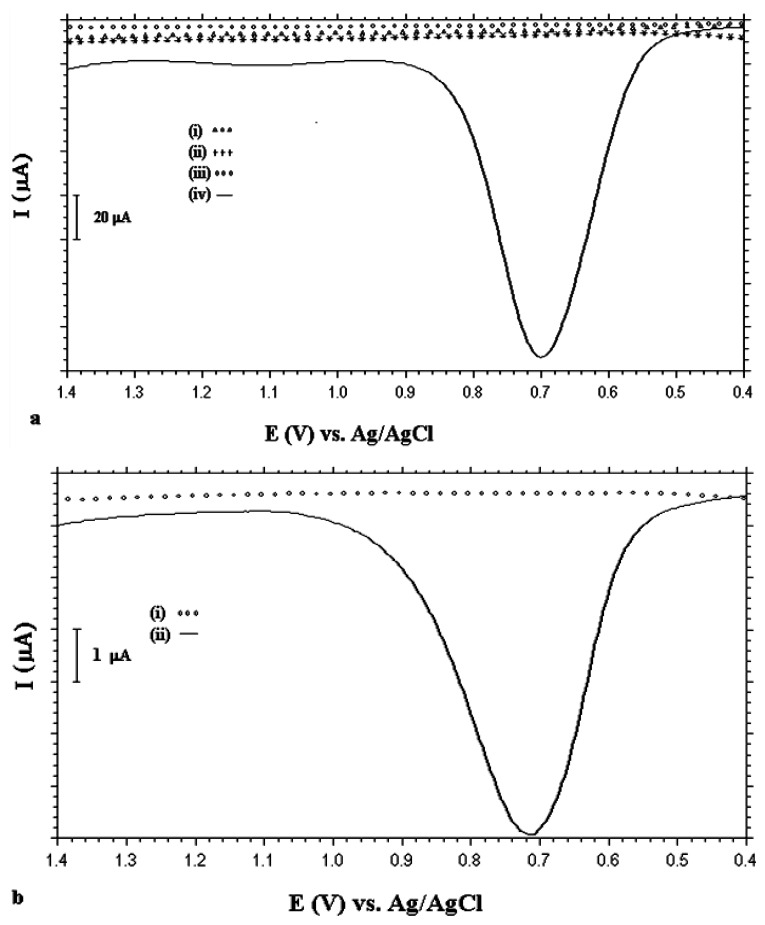
DPVs of (**a**) (i) PANI deposited Au electrode (ii) dsDNA immobilized PANI deposited electrode (iii) UME-2 (iv) dsDNA immobilized UME-2 (**b**) (i) ssDNA immobilized PANI deposited electrode (ii) ssDNA immobilized UME-2 in 50 mM PBS. *DPV measurement*: at pulse amplitude of 50 mV between +0.4 and 1.4 V.

Application of the formed UME was carried out using the PANI deposited (by the CV method) MUA-coated Au surface (UME-2) for DNA immobilization. For this purpose ds/ssDNA was immobilized onto UME-2. DPVs of PANI-deposited Au electrode, dsDNA immobilized PANI-deposited electrode, UME-2 and dsDNA immobilized UME-2 are given in Figure 11(a). As seen from this figure, for the PANI deposited Au electrode (Figure 11(a-i)) and UME-2 (Figure 11(a-iii)), there were no electroactive species between +0.4 V and 1.4 V *vs.* Ag/AgCl. With dsDNA immobilization onto the PANI-modified Au electrode, there was a very small shoulder at +1.1 V (Figure 11(a-ii)). After dsDNA immobilization onto the UME-2, the DPV in Figure 11(a-iv) was obtained. Enhanced electrochemical signals that belong to electroactive DNA bases, guanine and adenine were obtained in the case of UME-2. The peak at +0.7 V was attributed to guanine oxidation and the small peak at +1.1 V was attributed to adenine oxidation [42–45].

DPVs of the ssDNA-immobilized PANI-deposited electrode and ssDNA-immobilized UME-2 are given in Figure 11(b). As seen from this figure with ssDNA immobilization onto UME-2, a well developed guanine oxidation peak (Figure 11(b-ii)) was obtained compared to the PANI-deposited Au electrode (Figure 11(b-i)). Also, it can be claimed that the DNA oxidation signals were obtained in higher peak currents with dsDNA than with ssDNA (Figures 11(a-iv) to 11(b-ii)). Thus, the discrimination of dsDNA and ssdNA were carried out sensitively using UME-2. The ratio of the peak areas was calculated as 16.5. The dramatic increase in the guanine signal observed with dsDNA immobilized onto the polymer-modified electrode can be related to a much more availability of oxidation of guanine bases in dsDNA and electrostatic interactions between the cationic polymer structure and anionic DNA. The peak broadening can be attributed to the change in the conductivity of the polymer film after different polymerization conditions. The number of electrons was calculated for Figure 11(b-iv) (calf thymus dsDNA modified UME-2; calf thymus dsDNA contains 20% guanine) and was found to be 1.6 ± 0.2 electrons/guanine. This value was found as 2.2 ± 0.4 electrons/guanine by Armistead and Thorp [46]. Molar concentration, Gamma, of guanines immobilized on the surface (moles/cm^2^) was determined by using the equation: Gamma = Q/(n × F × A), where n is the number of electrons per guanine oxidation, F is Faraday's constant, and A is the surface area of the electrode. Electrically accessible molar quantity of guanine, Gamma, on the electrode surface was obtained as 4.51 × 10^−9^ mol·cm^−2^ and the molar quantity of DNA was found as 2.25 × 10^−8^ mol·cm^−2^.

The reproducibilities of the electrochemical responses of dsDNA and ssDNA immobilized electrodes were illustrated by five continuous experimental cycles. The results showed that dsDNA immobilized electrode had good reproducibility with a relative standard deviation of 7.9% (n = 5) and ssDNA immobilized electrode had good reproducibility with a relative standard deviation of 2.1% (n = 5).

**Figure 12.**
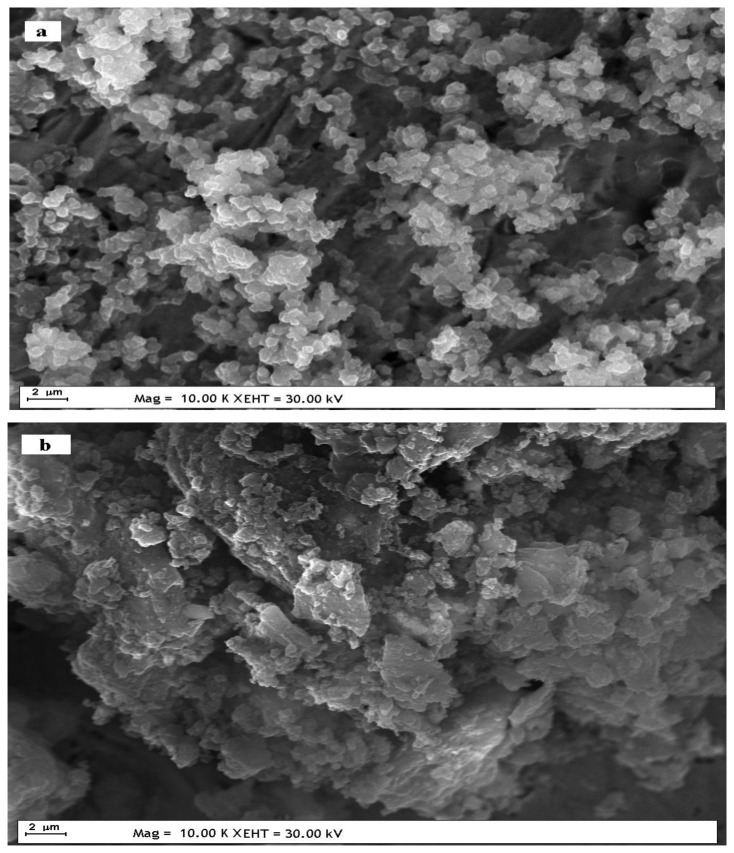
SEM images of (**a**) dsDNA immobilized UME-2 (**b**) ssDNA immobilized UME-2.

The surface morphologies of the dsDNA immobilized UME-2 and ssDNA immobilized UME-2 were examined using SEM (Figure 12). SEM images showed that some parts of the polymer film (Figure 10) were corrupted by dsDNA immobilization (Figure 12(a)) and ssDNA immobilization (Figure 12(b)). As a result, the absence of a PANI peak in the DPV voltammograms can be related to this drastical morphology change due to entrapment of DNA in the polymer matrix.This result is in agreement with the literature [42]. It can be deduced that ds DNA was immobilized on the surface more homogenously than ss DNA and thus revealed higher peak currents in the DPV curve.

## 4. Conclusions

Preparation of PANI-deposited 4-mercapto-1-butanol (MB)- and 11-mercaptoundecanoic acid (MUA)-modified Au surfaces was carried out in this study to form UMEs. The electrochemical behaviour of the obtained UMEs was investigated and compared with each other. The UME prepared with PANI deposited with cyclic voltammetry onto the MUA-modified Au surface showed the best performance.

Surface morphology of this UME examined by SEM showed the homogeneous distribution of PANI onto the electrode surface. In order to test the application of the formed UME, DNA immobilization was performed and enhanced electrochemical signals were obtained compared to the results obtained with only polyaniline-coated electrodes. Also, discrimination of dsDNA and ssDNA was performed. Surface morphologies of dsDNA and ssDNA-immobilized electrodes were examined using SEM. The improved sensitivity of the obtained UME suggests the possibility of further applications for specific DNA sequence sensing.

To the best of our knowledge, this is the first time, ultramicroelectrodes were prepared and characterized by electrochemical techniques and also applied for DNA discrimination. This method provides a cost effective way of UME formation when compared with the common lithographic methods. The obtained UME can lead to development of sensors for DNA detection.
